# Influence of selected environmental factors on the abundance of aerobic anoxygenic phototrophs in peat-bog lakes

**DOI:** 10.1007/s11356-016-6521-8

**Published:** 2016-04-01

**Authors:** Sylwia Lew, Marcin Lew, Michal Koblížek

**Affiliations:** Faculty of Biology and Biotechnology, University of Warmia and Mazury in Poland, Oczapowskiego 1a, 10-957 Olsztyn, Poland; Faculty of Veterinary Medicine, University of Warmia and Mazury in Poland, Oczapowskiego 2, 10-957 Olsztyn, Poland; Institute of Microbiology CAS, Center Algatech, 37981 Třeboň, Czech Republic

**Keywords:** Aerobic anoxygenic phototrophic bacteria, Peat-bog lakes, Humic and dystrophic lakes, Environmental factors, pH

## Abstract

Aerobic anoxygenic phototrophs (AAPs) are photoheterotrophic prokaryotes that are widespread in many limnic and marine environments. So far, little is known about their distribution in peat-bog lakes. Seventeen peat-bog lakes were sampled during three summer seasons 2009, 2011, and 2012, and the vertical distribution of AAPs was determined by infrared epifluorescence microscopy. The analysis demonstrated that in the surface layers of the studied lakes, AAP abundance ranged from 0.3 to 12.04 × 10^5^ cells mL^−1^, which represents <1 to 18.3 % of the total bacteria. The vertical distribution of AAPs confirmed their presence in the upper parts of the water column with minimum numbers in the anoxic bottom waters. We have shown that the AAP abundance was significantly positively correlated with the water pH, and the highest proportion of photoheterotrophs was found in peat-bog lakes with a pH between 6.7 and 7.6. Our results demonstrated an influence of water acidity on the abundance of AAPs, which may reflect a fundamental difference in the microbial composition between acidic and pH neutral peat-bog lakes.

**Electronic supplementary material:**

The online version of this article (doi:10.1007/s11356-016-6521-8) contains supplementary material, which is available to authorized users.

## Introduction

Aerobic anoxygenic phototrophs (AAPs) harvest light energy using bacteriochlorophyll *a* (BChl *a*) and auxiliary carotenoids. In contrast to many anoxygenic phototrophs, which grow under anaerobic conditions, AAPs are aerobic organisms which require oxygen for their metabolism and growth (Yurkov and Csotonyi 2009). In the euphotic zone of oceans, AAPs make up 1–10 % of the total bacterial population (Kolber et al. 2001; Sieracki et al. 2006; Jiao et al. 2007; Hojerová et al. 2011; Ritchie and Jonson 2012; Ferrera et al. 2014). These bacteria are also present in river (Ruiz-Gonzales et al. 2013), river estuaries (Waidner and Kirchman 2008; Cottrell et al. 2010), as well as brackish and saline lakes (Yurkova et al. 2002; Medová et al. 2011). AAPs were also found in a number of freshwater lakes (Mašín et al. 2008; Eiler et al. 2009; Martinez-Garcia et al. 2012; Caliz and Casamayor 2014; Fauteux et al. 2015). A few studies have shown that their proportion decreases with an increase in the lake trophic state (Jiao et al. 2007; Hojerová et al. 2011; Mašín et al. 2012). However, the relationship between trophic status and AAP abundance in freshwater systems is not clear yet. Apart from productive regions, light is an environmental factor, which can influence the number of bacterioplankton of AAP. A positive effect of light was also documented from seasonal studies conducted in the coastal waters and lagoons showing a positive correlation of AAP numbers and day length (Ferrera et al. 2014; Koblizek 2015). Another environmental factor which stimulates the growth of AAPs is temperature (Masin et al. 2006; Ferrera et al. 2014; Lew et al. 2015; Koblizek 2015). However, in ultraoligotrophic cold high mountain lakes, conductivity, pH, and nitrate concentration were main factors influencing the distribution and growth of AAP communities (Caliz and Casamayor 2014).

To date, AAPs have been sought in many different limnic ecosystems. A previous study of phototrophic community composition in Swedish lakes indicated that AAPs may prefer humic or dystrophic lakes (Eiler et al. 2009). However, the information on the presence of AAPs in these lakes is only limited (Mašín et al. 2012; Lew et al. 2015). Peat-bog lakes are small, shallow (up to 10 m deep) lakes with a low abundance of basic nutrients resulting in low productivity. They have a typical dark color, due to high concentration of humic substances originating from decomposing sphagnum plants surrounding the lakes. The dark color reduces the light penetration through the water column and causes an early establishment of thermal and oxygen stratification (Górniak et al. 1999). Stratification of the water column generates sharp physicochemical gradients which have a strong impact on microbial communities inhabiting the peat-bog lakes (Yannarell et al. 2003). The presence of humic acids increases the water acidity, which contributes yet to another factor affecting the composition of local microbial communities (Lindsröm 2000; Lindström et al. 2005), which is also due to the photoheterotrophic metabolism and participation of AAPs in the community.

AAPs are metabolically active organisms with a rapid growth, contributing significantly to the cycling of dissolved organic carbon (DOC) (Koblížek et al. 2007; Ferrera et al. 2011). The capacity of AAPs to use light energy increases their organic matter utilization efficiency, which indicates a unique role of AAPs in the microbial food webs (Koblížek 2011).

Carbon storage is an important ecological function of peat-bog ecosystems. In dystrophic lakes, bacterial and photochemical carbon mineralization processes are responsible for up to 70 % of the total carbon dioxide production in the water column (Jansson et al. 2001).

As photoheterotrophic bacteria use solar energy for effective utilization of organic matter, it can be assumed that AAPs may play a unique role in the microbial carbon pump. Therefore, it can be concluded that the presence of these bacteria in peat-bog ecosystems, where carbon uptake is an important ecological function, should be substantial. On the other hand, a low pH or a dark color of water can limit the presence of the AAP bacteria in peat-bog lakes, because growth and activity of microbial phototrophs might be affected by light extinction along the water column. In addition, these microorganisms have been found in large numbers in water at pH close to neutral. Moreover, the specific photoheterotrophic metabolism of bacteria can respond to environmental factors differently than the rest of bacterioplankton. In order to test the hypothesis that AAPs are more abundant in dystrophic lakes, the research was conducted in the summer, when the lakes provide good thermal and light conditions for the development and intensive proliferation of bacterioplankton.

## Material and methods

### Study sites and sampling

**Fig. 1:**
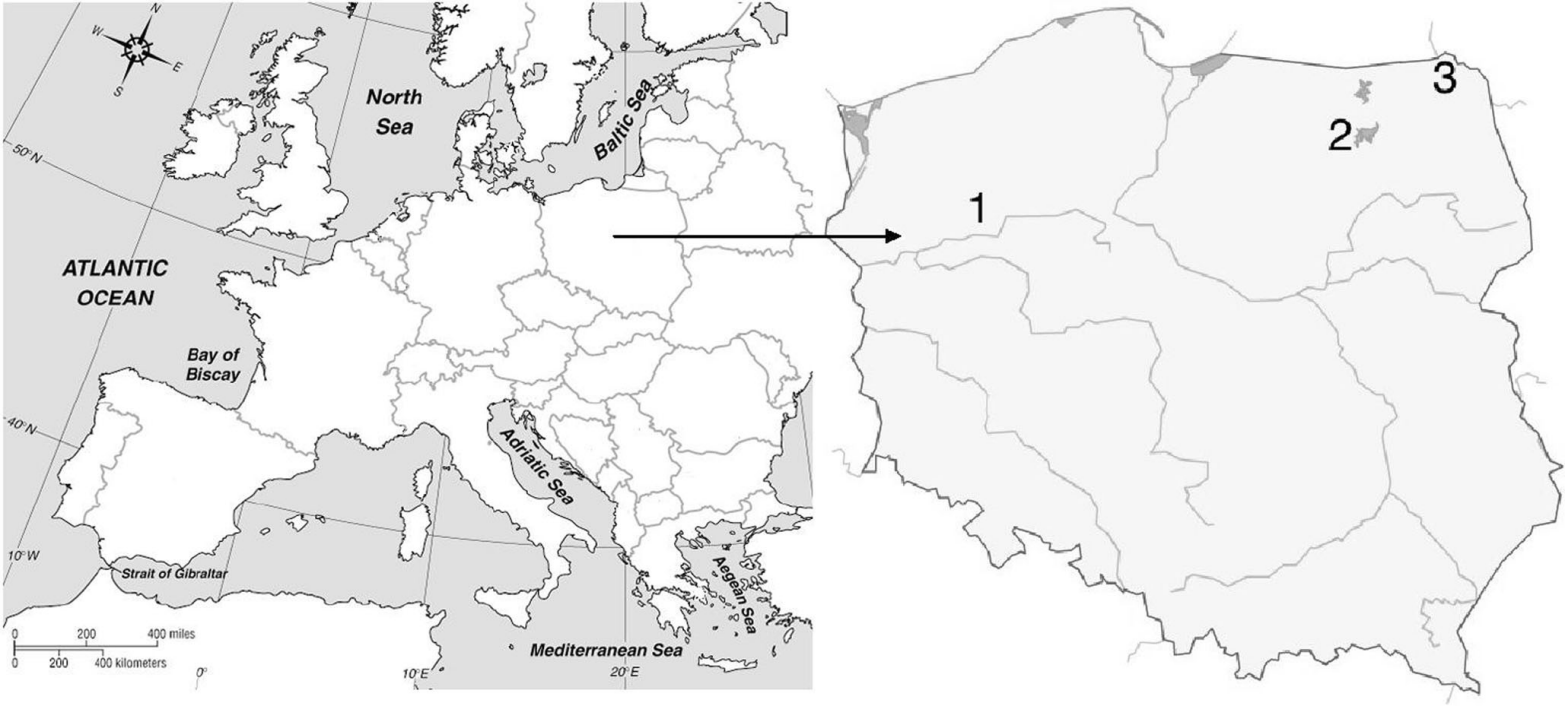
The three studied lake regions in Poland, Central-Eastern Europe

The study was conducted on 17 natural peat-bog lakes (Table 1) located in three regions of Poland, Europe (Fig. 1). Six lakes (Kuźniczek, Kuźnik Bagienny, Kuźnik Olsowy, Kuźnik Czarny—Czapla, Kuźnik Duży, and Kuźnik Mały) were selected in the Kuźnicke lake tunnel valley located in Wielkopolska region, mid-west Poland. Seven peat-bog lakes (Kruczki Kocie, Klimontek, Smolak Duży, Smolak Mały, Zakręt, Lwie Bagno Duże, and Lwie Bagno Małe) were chosen in the Masurian Lake District in north-east Poland. The other four were dystrophic lakes in Wigry National Park located in north-eastern Poland: Suchar 2, Suchar 3, Schar 4, and Wygorzele (Ślepiec). The samples were collected in summer periods (July–August) 2009, 2011, and 2012. The studied lakes were shallow waterbodies surrounded by a peat moss zone composed of different *Sphagnum* species. They were shadowed by dense pine stands. The lakes had a darker or brown water color and a layer that is well oxygenated and warm, with a shallow euphotic zone. The water samples were collected from a depth of 0.15 m in the central part of each peat-bog lake. In the case of the two deeper lakes, Suchar 2 and Suchar 4, samples were also collected from deeper layers, one determined by the visibility of a Secchi disk and the other above the bottom (1.8 and 9 m for Suchar 2, and 0.8 and 7.5 m for Suchar 4, respectively). Samples were taken three times, from all the lakes during each year of the study, and a total of 165 samples were collected. In each test, three replicates were performed for each single test.

**Table 1:** Location and morphometric data of studied lakes

Lake	Lake area (ha)	Max. depth (m)	Latitude and longitude
Kuźniczek^a^	0.04	2.0	53° 11′ 50″ N 16° 44′ 30″ E
Kuźnik Bagienny^a^	0.76	2.0	53° 12′ 52″ N 16° 43′ 52″ E
Kuźnik Czarny (Czapla)^a^	0.94	6.0	53° 13′ 41″ N 16° 43′ 56″ E
Kuźnik Duży^a^	0.997	2.0	53° 11′ 40″ N 16° 44′ 26″ E
Kuźnik Mały^a^	1.30	2.0	53° 11′ 50″ N 16° 44′ 21″ E
Kuźnik Olsowy^a^	0.37	1.0	53° 12′ 43″ N 16° 43′ 38″ E
Klimontek^b^	0.37	2.2	53° 42′ 27″ N 21° 36′ 28″ E
Kruczki Kocie^b^	0.43	9.0	53° 39′ 35″ N 21° 24′ 43″ E
Lwie Bagno Duże^b^	0.65	6.0	53° 45′ 41″ N 21° 28′ 42″ E
Lwie Bagno Małe^b^	0.20	2.0	53° 41′ 35″ N 21° 24′ 40″ E
Smolak Duży^b^	9.50	2.2	53° 43′ 27″ N 21° 36′ 09″ E
Smolak Mały^b^	3.50	3.0	53° 42′ 27″ N 21° 36′ 28″ E
Zakręt^b^	0.39	3.0	53° 41′ 14″ N 21° 24′ 40″ E
Suchar II^c^	2.50	9.5	54° 05′ 14″ N 23° 01′ 03″ E
Suchar III^c^	0.33	3.0	54° 05′ 19″ N 23° 01′ 18″ E
Suchar IV^c^	1.15	8.0	54° 05′ 23″ N 23° 01′ 29″ E
Wygorzele^c^	2.00	2.5	54° 01′ 30″ N 23° 08′ 53″ E

^a^Wielkopolska lakes

^b^Masurian Lake District

^c^Wigry National Park lakes

### Physicochemical parameters

Temperature, dissolved oxygen (DO) concentration, water color, conductivity, and pH were recorded using a multi-parameter YSI 6600 probe (YSI Inc., Yellow Springs, USA) directly when sampling. The DOC samples were transported to the laboratory, homogenized, pre-filtered with Whatman GF/F filters, and the DOC concentration was determined using a Shimadzu TOC_V-CSH_ Total Organic Carbon Analyzer (Pan et al. 2005).

Chlorophyll *a* (Chl *a*) and BChl *a* concentrations were determined by HPLC according to a previously described protocol (Medová et al. 2011). Peat-bog water (from 0.5 to 2 L) was filtered under a low vacuum through two overlying GF/F filters that were frozen immediately after filtration at −80 °C until analysis. They were then homogenized in a 7:2 (vol/vol) mixture of acetone/methanol and centrifuged for 10 min at the speed of 6000 rpm. The clear extracts were analyzed by HPLC with an Agilent Technologies 1100 Series system. The instrument was equipped with the UV–VIS diode-array detector (Agilent DAD 61315B) and an in-line Agilent 1100 Series LC/MSD Trap mass spectrometer with APCI chemical ionization module (nebulizer gas 50 psi, nebulizer temperature 350 °C, capillary potential 4000 V, corona 4 A, vaporizer temperature 400 °C, freq. amplitude 1.5 V). Pigments were separated using a modified method of Van Heukelem and Thomas (2001) on the heated (35 °C) Phenomenex Luna 3 μ C8(2) 100 Å column with binary solvent system (0 min 100 % A, 20 min 100 % B, 25 min 100 % B, 27 min 100 % A, 30 min 100 % A; A: 70 % methanol + 28 mM ammonium acetate, B: methanol). The solvent flow rate was 0.8 mL min^−1^. The peak assignment was based on acquired absorption spectra and confirmed by in-line mass spectrometry. Chl *a* was detected at 660 nm; BChl *a* was detected at 770 nm. The detection limit for Chl *a* was approximately 50 pg per injection (Koblížek et al. 2010).

### Epifluorescence microscopy

The total number of bacteria (TNB) was determined using epifluorescence microscopy (Porter and Feig 1980). Triplicate subsamples (50 mL) were fixed with neutralized formaldehyde (pH 7.4) at a final concentration of 4 %. In the laboratory, the samples (1 mL) were stained with DAPI (final concentration 0.01 μg mL^−1^) for 15 min in the dark and gently filtered through 0.2-μm black nuclepore filters. The bacteria were counted using an Olympus BX41 epifluorescence microscope. More than 1000 bacterial cells were counted in 20 microscope fields.

The number of bacteriochlorophyll-containing bacteria was determined by infra-red (IR) epifluorescent microscopy (Mašín et al. 2006). The fixed samples (50 mL) were stored in a freezer (−20 °C). After thawing, the samples (2 mL) were collected onto 0.2-μm polycarbonate filters, dried, and stained with DAPI (1 μg mL^−1^) mixed with Citifluor AF1 and Vectashield (3:1 vol/vol). The cells were counted using the Olympus BX51TF fluorescence microscope with an Olympus Universal Planapochromat 100×/1.35 OIL objective equipped with a B/W CCD camera F-ViewII. Firstly, the total DAPI-stained bacteria were recorded in the blue part of the spectrum (100–200 ms exposure). Then, the IR emission (>850 nm) image was captured, showing both AAPs and phytoplankton (15–35 s exposition). Finally, red Chl *a* autofluorescence was recorded to identify Chl *a*-containing organisms (0.5–1 s exposition). The acquired images were saved and semi-manually analyzed with the aid of AnalySiS software (Soft Imaging Systems). The individual images were artificially colored (DAPI: blue; Chl *a*: green; IR: red—see Supplemental material) and overlaid to create a composite image. The composite image allowed us to distinguish the Bchl *a*- and Chl *a*-containing microorganisms and to obtain net counts of heterotrophic bacteria, picocyanobacteria, and AAPs for each sample. The objects were counted manually to avoid problems with automatic assessment. For each sample, ten fields of view were recorded and analyzed and more than 600 DAPI cells were counted per sample (Mašín et al. 2006).

### Statistical analyses

Lake samples were taken in triplicate to determine the variability of DAPI counts. The correlation analysis, mean abundances, and standard deviations (SDs) were calculated using Statistica v.8. The response of the microbiological communities to the environmental conditions was analyzed using multivariate statistical analyses. Detrended correspondence analysis (DCA) and redundancy analysis (RDA) were performed using CANOCO 4.5. The DCA of the microbiological parameters was used to determine whether linear or unimodal ordination methods should be applied (ter Braak and Šmilauer 2002). DCA was used first to determine the variability in the studied assemblages: if a gradient length was over 4 SD, species in the data show a clear unimodal response along the gradient. The gradient length amounted to SD = 0.054, which indicated a linear variation, providing justification for the further use of RDA, which is a direct gradient analysis that summarizes relations between bacteria and water quality parameters. The dataset was centered and standardized by species, due to the different units of environmental variables. To rank the importance of the individual explanatory variable, automatic forward selection of environmental variables was used. Before each addition, the explanatory effect of the candidate variable was evaluated using the Monte Carlo permutation test (Lepš and Šmilauer 2003).

## Results

The abundance of AAPs was studied in 17 lakes located in three different regions of Poland—Wielkopolska, Masurian Lake District, and Wigry National Park (Fig. 1). The three selected regions differ in annual temperatures, precipitation, and the duration of ice cover. Wielkopolska is characterized by milder winters and higher annual temperatures, whereas the coldest climate is found in Wigry National Park.

This study was conducted during the summer when the surface water temperatures exceeded 20 °C. Due to different climatic conditions, there were some small temperature differences between the regions. The lakes in Wigry National Park (*n* = 6) were on average 1.3 °C colder than the total average temperature (*T* = 23.6 °C), whereas the Wielkopolska and Masurian lakes were on average 0.1 and 0.7 °C warmer than the total average (Table 2).

**Table 2:** Main physicochemical characteristics of the studied lakes

Lake	Temperature (°C)	pH	Water color (mg Pt L^−1^)	DOC (mg C L^−1^)	Dis. oxygen (mg L^−1^)	Conductivity (μS cm^−1^)
Kuźniczek	24.84 ± 1.81	5.5 ± 0.17	30 ± 3.0	25.70 ± 3.81	4.16 ± 0.38	98.14 ± 5.54
Kuźnik Bagienny	26.62 ± 1.96	6.69 ± 0.32	76 ± 4.0	28.21 ± 1.91	5.14 ± 0.3	430.76 ± 26.27
Kuźnik Czarny	24.24 ± 1.65	7.1 ± 0.30	19 ± 1.5	14.7 ± 2.74	7.97 ± 0.38	325.39 ± 10.90
Kuźnik Duży	21.41 ± 0.87	7.23 ± 0.38	43 ± 3.5	6.67 ± 2.07	7.60 ± 0.16	417.32 ± 14.71
Kuźnik Mały	21.68 ± 1.10	7.33 ± 0.34	30 ± 2.5	9.47 ± 1.59	7.68 ± 0.50	519.08 ± 30.92
Kuźnik Olsowy	23.14 ± 1.41	7.02 ± 0.11	58 ± 2.2	21.88 ± 3.40	6.74 ± 0.40	525.77 ± 25.80
Klimontek	24.90 ± 1.77	7.37 ± 0.45	80 ± 3.8	8.41 ± 2.01	11.52 ± 1.74	100.22 ± 9.28
Kruczki Kocie	24.59 ± 1.45	4.75 ± 0.19	195 ± 9.5	3.62 ± 1.23	11.50 ± 2.14	177.00 ± 19.45
Lwie Bagno Duże	24.11 ± 1.80	7.52 ± 0.34	32 ± 2.0	4.70 ± 0.77	11.22 ± 2.45	158.41 ± 4.32
Lwie Bagno Małe	24.64 ± 1.58	8.41 ± 0.46	18 ± 1.0	4.04 ± 1.29	16.11 ± 3.85	116.88 ± 14.93
Smolak Duży	20.88 ± 1.18	4.60 ± 0.22	549 ± 22.8	40.29 ± 10.20	8.65 ± 0.74	39.42 ± 4.88
Smolak Mały	24.55 ± 2.07	3.57 ± 0.15	1820 ± 30.5	103.15 ± 7.10	8.11 ± 0.56	79.05 ± 8.36
Zakręt	26.14 ± 1.41	4.94 ± 0.24	149 ± 7.0	3.10 ± 0.93	12.89 ± 1.92	216.32 ± 14.82
Suchar II	23.77 ± 1.21	6.01 ± 0.58	85 ± 4.5	8.66 ± 1.67	8.55 ± 0.20	17.72 ± 4.26
Suchar III	21.79 ± 1.25	4.62 ± 0.50	117 ± 9.5	13.38 ± 1.69	8.02 ± 0.29	21.31 ± 1.21
Suchar IV	22.34 ± 1.47	4.5 ± 0.39	199 ± 11.5	20.18 ± 4.82	8.19 ± 0.43	24.34 ± 4.84
Wygorzele	21.12 ± 0.99	4.72 ± 0.11	242 ± 12.5	19.87 ± 3.22	8.15 ± 0.37	20.08 ± 3.64

Provided values are means ± st. dev. of samples collected in summer 2009, 2011, and 2012

*DOC* dissolved organic carbon concentration

**Fig. 2:**
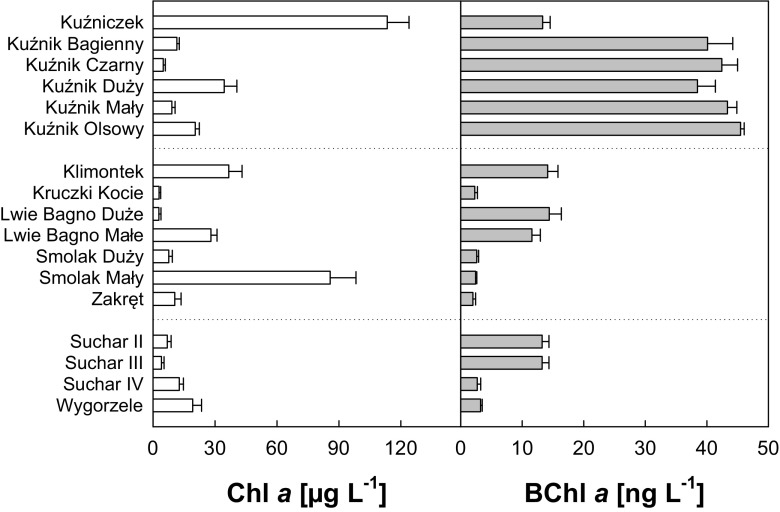
Chl *a* and BChl *a* concentration in peat-bog lakes during the summers of 2009, 2011, and 2012

**Fig. 3:**
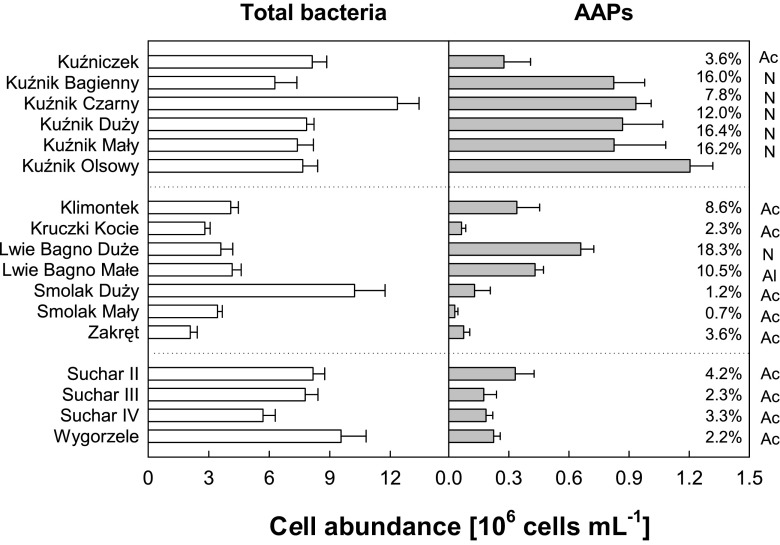
Average values of abundance of AAPs and TNB in lakes during the summers of 2009, 2011, and 2012 (*Ac* (*acidic lakes*): Kuźniczek, Kruczki Kocie, Klimontek, Smolak Duży, Smolak Mały, Zakręt, Suchar 2, Suchar 3, Suchar 4, and Wygorzele (Ślepiec); *N* (*neutral lakes*): Lwie Bagno Duże, Kuźnik Bagienny, Kuźnik Olsowy, Kuźnik Czarny—Czapla, Kuźnik Duży, and Kuźnik Mały; *Al* (*alkaline lake*): Lwie Bagno Małe)

The studied lakes were selected to cover a broad range of physicochemical parameters (Table 2) reflecting different trophic conditions and different stages of succession between dystrophy and eutrophy (Gąbka and Owsianny 2006). The average pH ranged between 3.6 and 8.4 (Table 2). The lowest pH was found in the dark brown lake Smolak Mały, whereas the highest pH was in the slightly alkaline pool called Lwie Bagno Małe (Table 2). The DOC concentration varied between 3 and 110 mg C L^−1^. In most of the lakes, the DOC concentrations were below 20 mg C L^−1^, with the lowest concentrations found in the Masurian peat-bog lakes (Table 2). The highest DOC concentrations were found in Smolak Duży and Smolak Mały, with 40.29 and 103.15 mg C L^−1^, respectively (Table 2). The statistical analysis showed a slight negative correlation between DOC concentration and water pH (*r* = −0.5569, *p* < 0.05). Water conductivity varied from 20.08 to 525.77 (μS cm^−1^). The highest values were detected in lakes with neutral pH (pH 6.69–7.02): Kuźnik Bagienny, Czarny, Duży, Mały, and Olsowy (Table 2). Similarly to large differences in physicochemical variables, the investigated lakes also differed in the main microbial variable. The Chl *a* concentration largely varied from 2.8 up to 113 μg Chl *a* L^−1^ found in the highly trophic lake Kuźniczek (Fig. 2). A positive correlation was observed between Chl *a* concentration and DOC concentration. The total bacterial numbers (TBNs) ranged from 2.09 to 12.35 × 10^6^ cells mL^−1^ (Fig. 3).

**Fig. 4:**
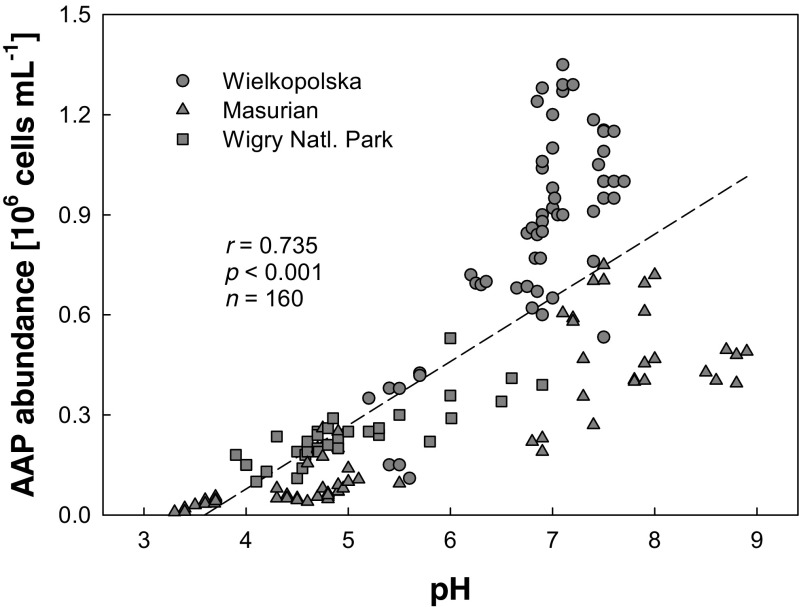
Relationship between the water pH and AAP abundance in lakes sampled during 2009, 2011, and 2012

**Fig. 5:**
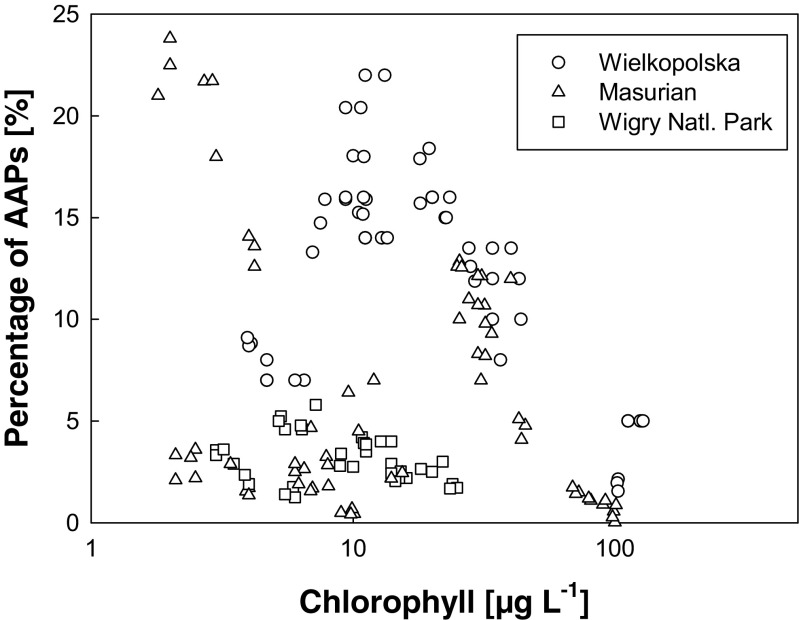
Relationship between the chlorophyll concentration and AAP percentage in the studied lakes during the summers 2009, 2011, and 2012

The highest AAP numbers were recorded in neutral or slightly alkaline waterbodies in the Wielkopolska region (Fig. 3). In acidic lakes such as Smolak Mały and Zakręt, the AAP abundance was low, less than 1 % of the TBN. This phenomenon can be documented by a strong positive correlation between AAP abundance and water pH (see Fig. 4) as well as between AAP abundance and conductivity (Table 3). The percentage of AAPs in the total bacterioplankton ranged from less than 1 to more than 18.3 % with higher numbers observed in lakes with low values defining the color of the water (mg Pt L^−1^) and lower Chl *a* concentrations (Fig. 5); however, the statistical correlations were only weak (Table 3). Similarly to AAP abundance, BChl *a* concentration was strongly correlated to water pH and conductivity (Table 3). On the other hand, no significant correlations were found between AAP abundance or BChl *a* concentration and chlorophyll concentration, or water temperature.

**Table 3:** Pearson product-moment correlation coefficients between AAP abundance, percentage of AAPs, and BChl *a* concentration and various physicochemical parameters calculated for 160 individual samples

Variables	AAPs		% AAPs		BChl *a*	
	*r*	*p*	*r*	*p*	*r*	*p*
BChl a*	0.921	0.000	0.741	0.000	–	–
Chl *a*	−0.201	0.011	−0.243	0.002	−0.132	0.095
TBN	0.313	0.000	−0.079	0.324	0.357	0.000
Lake area	−0.298	<0.001	−0.370	<0.001	−0.283	<0.001
Temperature	−0.048	0.546	0.074	0.354	0.039	0.621
pH*	0.735	0.000	0.796	0.000	0.650	0.000
DOC	−0.280	<0.001	−0.339	<0.001	−0.193	0.014
Oxygen	−0.258	<0.001	0.003	0.971	−0.378	<0.001
Conductivity*	0.735	0.000	0.668	0.000	0.815	0.000
Water color	−0.442	0.000	−0.440	0.000	−0.377	0.000

Strong positive correlations are marked in *

*r* correlation coefficient, *p* probability, *DOC* dissolved organic carbon concentration, *oxygen* dissolved oxygen concentration

A very strong positive correlation was found between the AAP abundance and BChl *a* concentration (Table 3), which indicates that most of the measured BChl *a* came from AAP species.

**Fig. 6:**
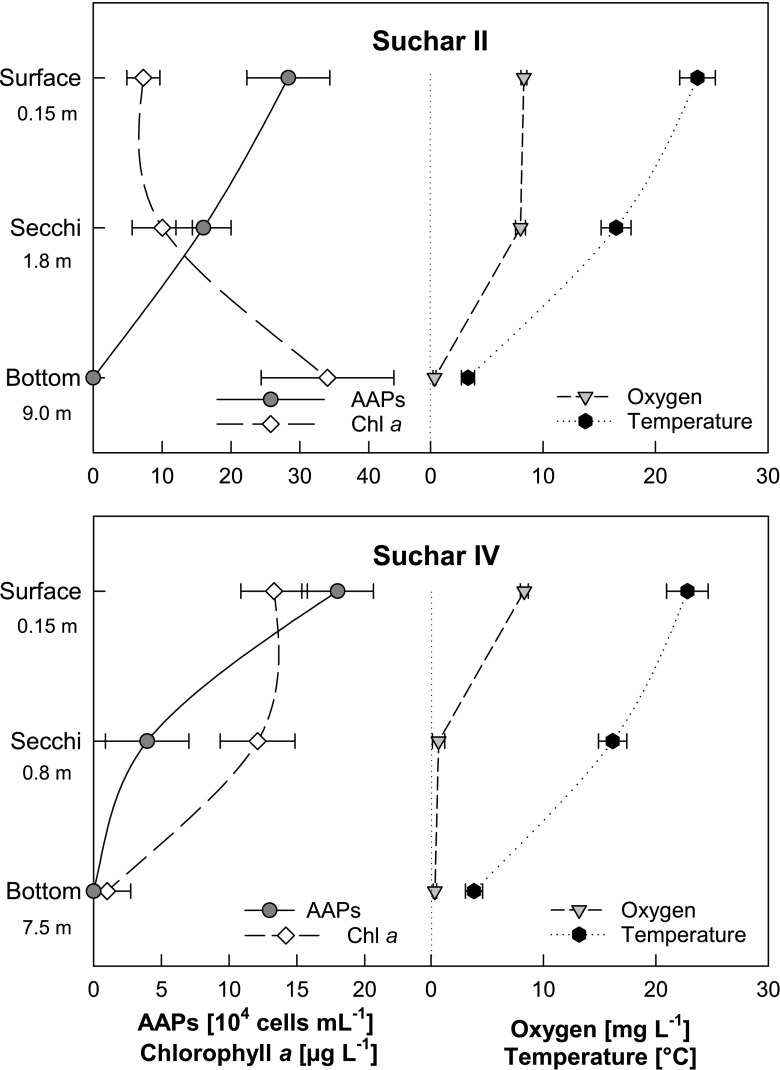
Depth profile of the average value (mean; error bars ± SD) of abundance of AAPs, chlorophyll a concentrations (*Chl a*), temperature (*T*), and oxygen from Suchar 2 and Suchar 4

To get further insights about AAP distribution, we decided to measure a water column profile of two deeper systems: Suchar 2 and Suchar 4 (Fig. 6). As expected, the AAP bacteria were mostly present in the upper oxic water layers, whereas in deeper layers, their numbers declined. In mostly anoxic bottom waters, the AAP cells were not observed. The AAP abundance strongly correlated in both lakes with oxygen concentration and water temperature. This suggests that the observed BChl *a*-positive cells were indeed AAP species and not anaerobic phototrophs such as green sulfur bacteria, which were reported from Finnish peat-bog lakes (Karhunen et al. 2013).

**Fig. 7:**
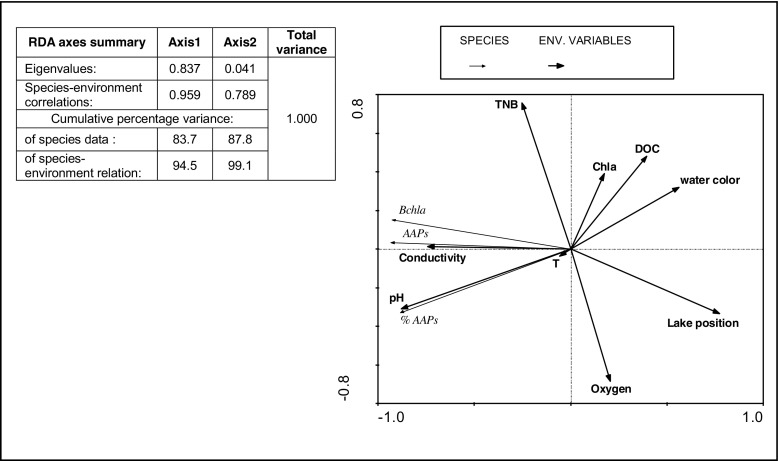
Ordination plot of redundancy analysis (RDA) for AAPs, AAPs%, BChl *a* (*species*), and TNB with hydrochemical data (*environmental variables*) for study lakes

RDA was used to explore the relationships between the abundance of AAPs and environmental variables. Forward selection in RDA identified eight environmental variables (pH, temperature, conductivity, water color, DOC, oxygen, TNB, lake position, and Chl *a*) that explained significant (*p* ≤ 0.05), independent directions of variation in the microbiological data (%AAPs, AAPs, and BChl *a*) from lakes (Fig. 7). RDA performed for microbiological data and environmental parameters showed that the first and second axes explained 94.5 and 99.1 % of the cumulative variance, respectively (Fig. 7). Of the explanatory environmental variables, pH, conductivity, and water color had the strongest relationships to the primary axis explaining 0.66, 0.03, and 0.01 of variance, respectively. Oxygen and TNB had the strongest relationships to the second axis explaining 0.16 and 0.01 of variance, respectively (Table 4). The environmental variables contributed significantly to the model of already included variables (after Monte Carlo permutations) at *p* = 0.002. Among microbiological variables, AAPs showed a significant gradient related to the first axis.

**Table 4:** The explanatory variables selected that represent significant relation between species (marginal and conditional effects)

Variable	Marginal effects	Conditional effects		
	λ1	λA	*p*	*F*
pH	0.66	0.66	0.002	303.64
Conductivity	0.46	0.03	0.002	26.66
Water color	0.27	0.01	0.002	4.06
DOC	0.14	0.00	0.010	5.37
TNB	0.08	0.01	0.002	17.20
Oxygen	0.05	0.16	0.002	147.65
Chl *a*	0.03	0.01	0.004	8.24
Temperature	0.01	0.00	0.018	4.41
Lake position	0.40	0.01	0.134	1.95

Lake position corresponds to the lakes’ longitude

## Discussion

It is well established that the environmental factors such as concentration of nutrients, pH, or temperature have a profound impact on the freshwater microbial community composition and diversity (Muylaert et al. 2002; Stepanauskas et al. 2003; Lindström et. al. 2005; Caliz and Casamayor 2014). Our research on peat-bog lakes confirms that even in these ecosystems, selected environmental factors regulate the participation in the community of aerobic anoxygenic phototrophs. The analyses demonstrated that AAP abundance is positively correlated with pH: the highest absolute and relative abundance of AAP was recorded for the lakes with neutral pH. In acidic lakes with pH in the surface layer below 4.9, AAPs constituted less than 1 % of the TNB.

The effect of water pH on bacterial community is a well-known phenomenon (Lingstrom et al. 2005; Yannarell et al. 2005; Fierer and Jackson 2006; Percent et al. 2008; Lin et al. 2012). The pH significantly affects the bacterial community composition (Lin et al. 2012) and positively influences the species diversity and richness (Percent et al. 2008). In low-pH ecosystems, smaller quantities of microorganisms are recorded and selectivity for acid-tolerant phylotypes is observed (e.g., Acidobacteria), while under more neutral pH conditions, a wide variety of phylotypes is observed (Lin et al. 2012; Sait et al. 2006).

The reason for the overall low numbers of AAPs at low pH is not clear. It does not seem that the low numbers would be caused by the acidic conditions itself as there exist a number of acidophilic AAP species belonging to the genus *Acidiphilium* which thrive in the pH range of 2.5 to 5.9 (Hiraishi and Shimada 2001). *Acidiphilium*-related sequences were detected in environmental DNA samples collected from the north-west sector of highly humic Lake Grosse Fuchskule, Germany (Salka et al. 2011), which indicates that these species are present in European humic lakes. Moreover, highly abundant AAP communities were reported from acidified mountain lakes Plešné, Černé a Čertovo in Šumava mountains, Czech Republic (Mašín et al. 2008). In mesotrophic mountain lake Plešné (pH∼5.1), AAP bacteria formed, during summer, over 50 % of bacterial biomass with BChl *a* concentrations reaching up to 400 ng BChl *a* L^−1^, which is the highest density of AAP bacteria ever reported for a freshwater lake (Mašín et al. 2008). These facts indicate that the observed relationship between pH and AAP abundance is not universally valid in all freshwater habitats and applies only to our dataset of peat-bog lakes sampled during summer season. The AAP abundance may also have been influenced by other factors, which may be directly or indirectly affected by water chemistry and pH. The water pH may also significantly alter the composition of protistan and zooplankton grazers. It has been documented that AAPs are regularly under intense grazing pressure (Ferrera et al. 2011), and a change of grazer community may significantly affect AAP numbers.

Similarly to the effect of pH, we also found a significant correlation between conductivity and AAP abundance. A strong positive correlation of conductivity with the bacterioplankton community composition has already been reported earlier (Lindström 2000; Allgaier and Grossart 2006; Caliz and Casamayor 2014). This relationship is most likely the result of a positive correlation between conductivity and pH (*r* = 0.526756, *p* < 0.05). The charge of mineral compounds present in water, whose determinant is conductivity, creates neutral mineral—humus compounds with humic acids penetrating into water; thus, the acidity of the reservoir, where there has been a higher proportion of AAPs in the community of microorganisms, is reduced.

The statistical analysis also indicated a negative correlation of AAP abundance and water color. This relationship is not easy to interpret. One possible explanation is that the darker lakes are usually those with a low pH, so the relationship between water color and AAP abundance is only indirect. The second possibility is that AAPs as photoheterotrophic organisms may prefer more transparent lakes with better light penetration. However, the fact that we did not observe such correlation during our previous peat-bog lake study (Lew et al. 2015) makes this option less likely.

Interestingly, the presented data did not show any positive correlation between AAP abundance and Chl *a* concentration. The negative relationship between AAPs and Chl *a* was also discovered by Ferrera et al. (2014), which combined with information about increased presence of AAPs, and annual accumulation of dissolved organic carbon (DOC) which becomes chemically and structurally more complex may indicate that AAP bacteria rely on phototrophy as an auxiliary energy source. The positive correlation between AAPs and Chl *a* concentration was repeatedly reported from both marine (Sieracki et al. 2006; Hojerová et al. 2011; Lamy et al. 2011) as well as limnic environments (Medová et al. 2011; Mašín et al. 2012). In our recent study of seasonal changes of microbial community in two peat-bog lakes, we observed such a relationship only in Kuźnik Olsowy, whereas in Kuźnik Bagienny, the relationship was negative (Lew et al. 2015).

Another important factor affecting AAP growth is water temperature. The influence of temperature on AAP community has been repeatedly observed during seasonal studies conducted in both marine (Mašín et al. 2006; Ferrera et al. 2014) and freshwater systems (Mašín et al. 2008; Čuperová et al. 2013). The strong correlation between AAP abundance and temperature was also confirmed in our seasonal study of two peat-bog lakes in Poland (Lew et al. 2015). However, in the presented dataset, the effect of temperature has not been observed, probably due to the only minimal differences between surface water temperatures between the studied lakes.

In conclusion, AAPs are a common component of the microbial community in peat-bog lakes. The analysis demonstrated that in the surface layers of the studied lakes, AAP abundance ranged from 0.3 to 12.04 × 10^5^ cells mL^−1^. The vertical distribution of AAPs confirmed their presence in the upper parts of the water column with minimum numbers in the anoxic bottom waters. These organisms prefer pH neutral lakes (between 6.7 and 7.6) with higher conductivity and water transparency. During summer season, they may account for up to 20 % of total bacteria in these habitats. Our results demonstrated an influence of water acidity on the abundance of AAPs, which may reflect a fundamental difference in the microbial composition between acidic and pH neutral peat-bog lakes.

## Electronic supplementary material

Below is the link to the electronic supplementary material.

ESM 1 (DOCX 1051 kb)
