# Supplementary material for: Influence of selected environmental factors on the abundance of aerobic anoxygenic phototrophs in peat-bog lakes
Source: Environ Sci Pollut Res Int. 2016 Apr 1;23:13853–63. doi: 10.1007/s11356-016-6521-8 (PMC4943989; doi:10.1007/s11356-016-6521-8)
Supplement: Supplementary file 1 — (DOCX 1051 kb) [file 11356_2016_6521_MOESM1_ESM.docx]

A sample set of photos allowing the identification and counting of AAPs using the epifluorescence microscopy method

*fot. Michal Koblížek*


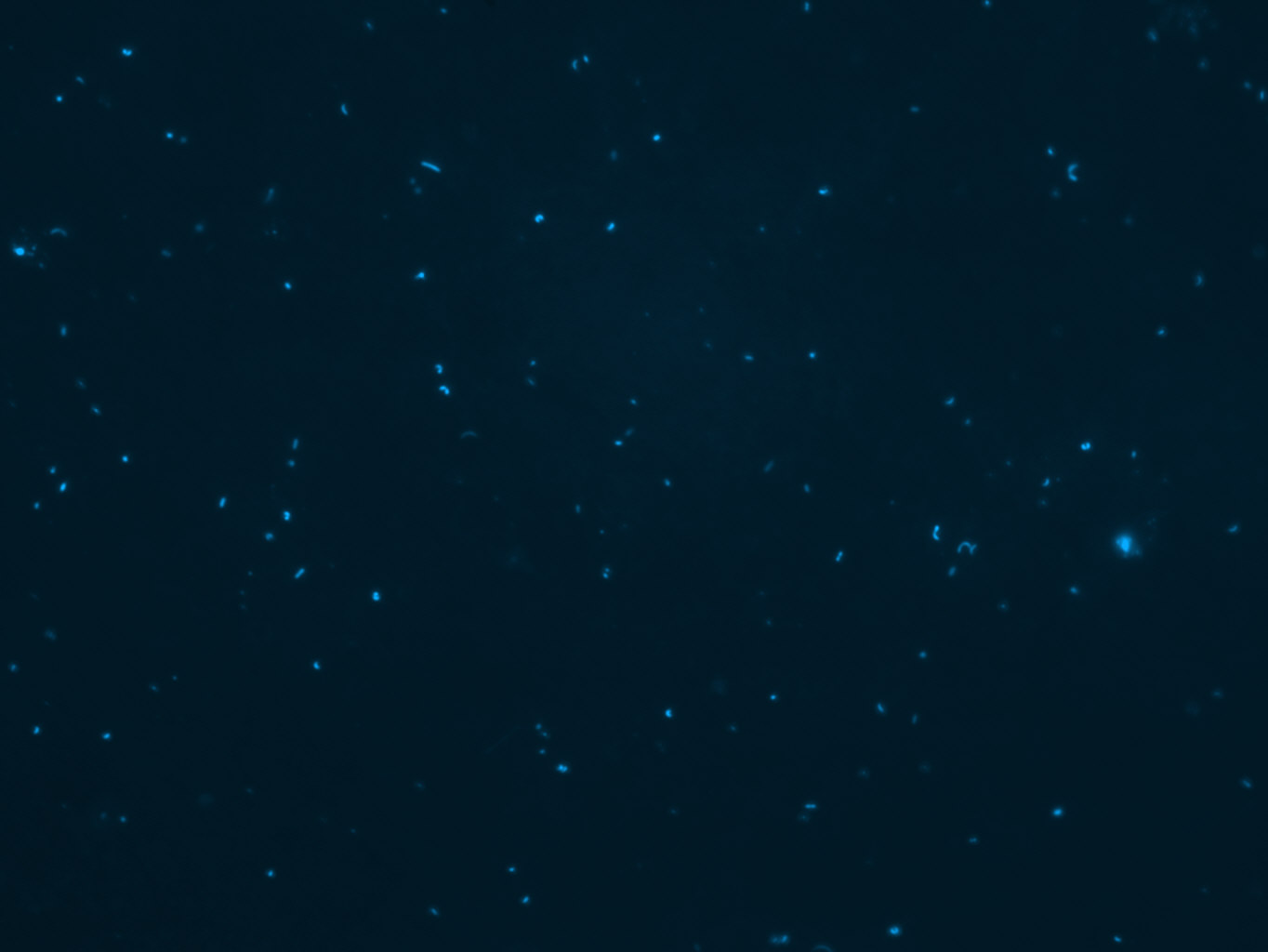

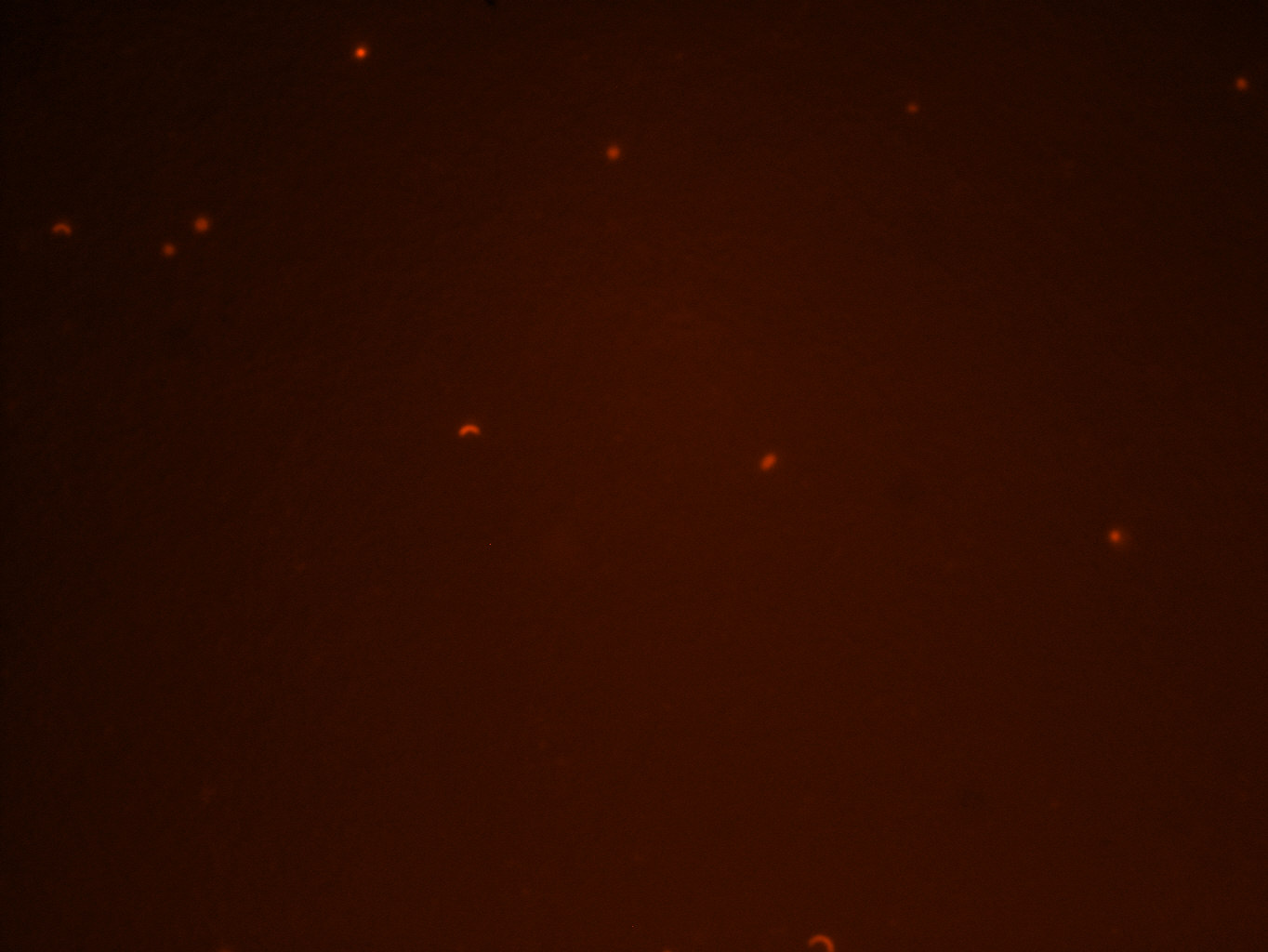

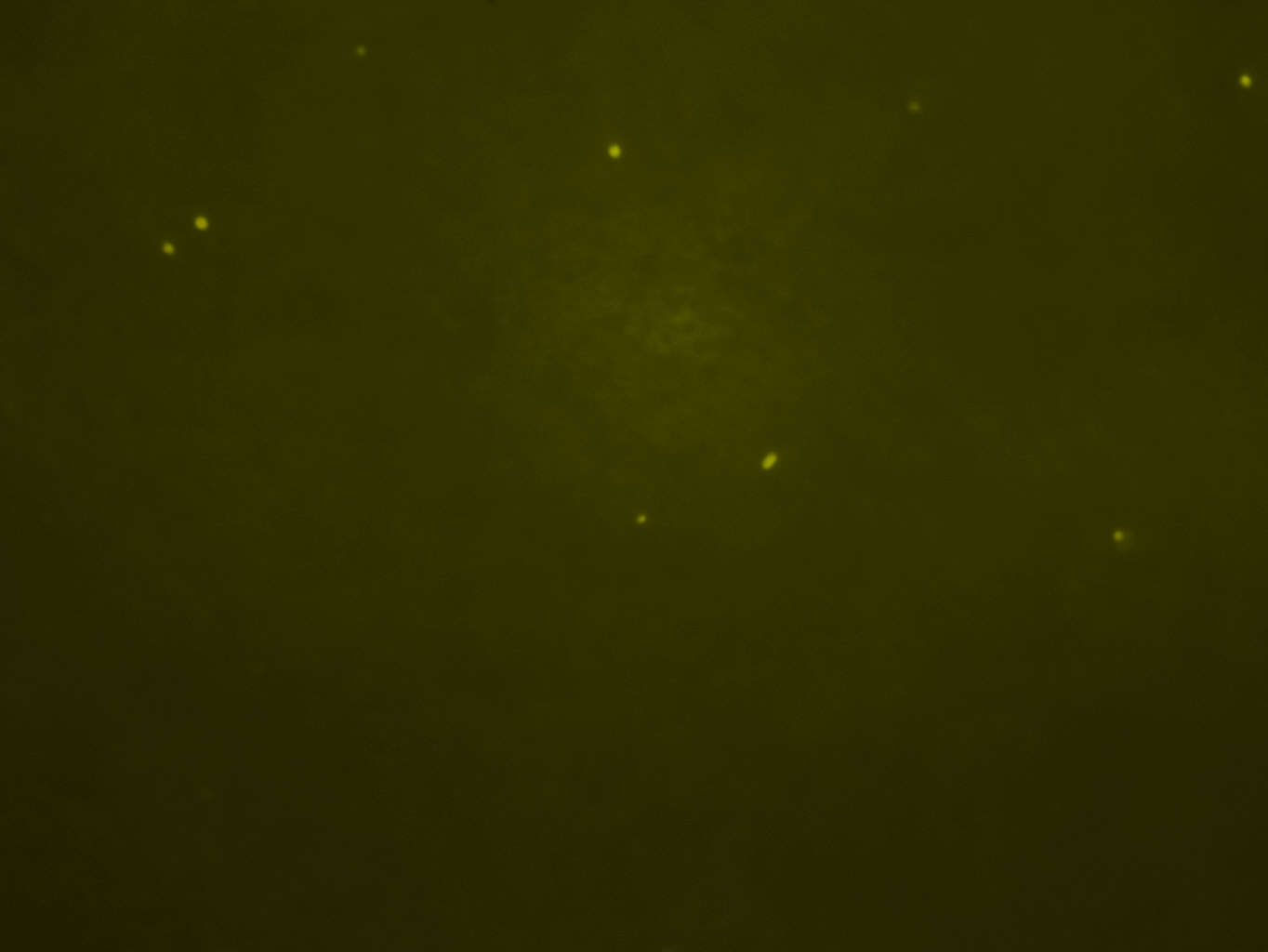


**DAPI**

**IR Bchl a** aBChla

Chl a


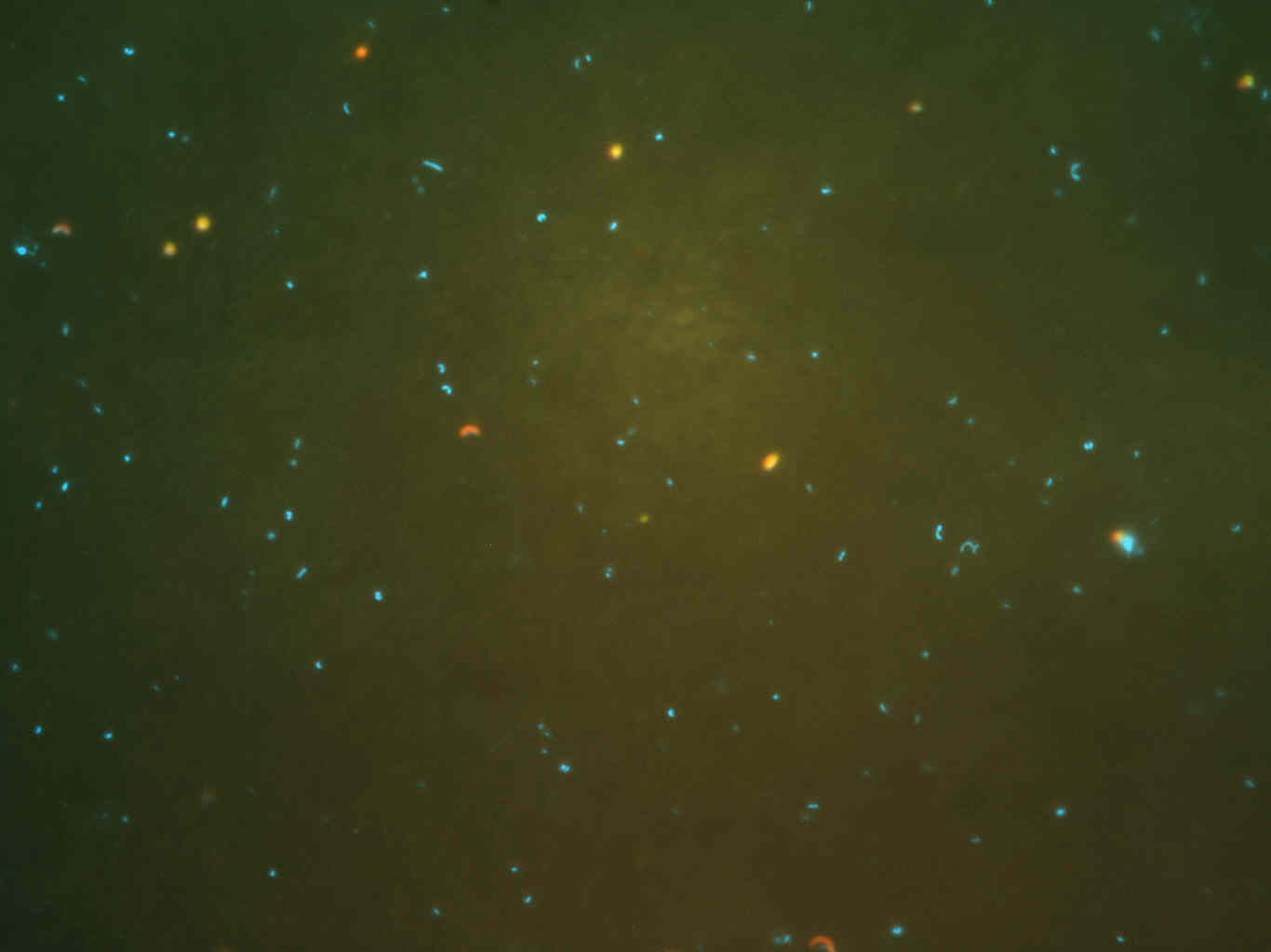


**Picocyanobacteria**

**AAPs**
